# Pulmonary Emphysema and Its Relation to Heaves in the Horse*From the Wochenschrift für Theirheilkunde uud Viehzucht.

**Published:** 1885-04

**Authors:** Wilhelm Dieckerhoff

**Affiliations:** Conductor of the Clinic of the Royal Veterinary School, Berlin


### Art. XIV.—PULMONARY EMPHYSEMA AND ITS RELATION TO HEAVES IN THE HORSE.*

BY PROF. WILHELM DIECKERHOFF,

(Conductor of the Clinic of the Royal Veterinary School, Berlin).

Whosoever, engaged in veterinary medicine, has taken pains to thoroughly study the pathology, the diagnosis and theories concerning the various diseases and abnormal conditions of the organs of respiration, will have noticed how defective our present knowledge of heaves is. Attempts to explain the causes of heaves have never been wanting. In reviewing the history of veterinary medicine, I must admit that some theories were advanced with considerable ingenuity. Perhaps we would have progressed more rapidly in diagnosing the various forms of heaves had it not been for the existence of laws regulating the buying and exchange of heavey horses, which necessitated the most eminent authors to admit of an extensive interpretation of the term heaves, in order to prevent the buyer from being imposed upon. With regard to existing special laws, entitling the buyer to return the horse and reclaim from the seller the purchase money if certain defects can be proven within the time stated by law, it cannot be denied that it would be practical to put numerous pulmonary conditions under the forensic term—heaves.

The definition, defended up to the present time, that heaves is a non-febrile, labored respiration, originated with Lafosse (Cours d'Hippiatrique, 1772), who, as well as his predecessors and immediate successors, located the trouble in the lungs. The proofs that chronic diseases of the heart caused labored respiration (heaves) were first produced by Dupuy and Godine, Jr. ("Elemens d'Hygiene Veterinaire, etc., 1815), whilst roaring was previously defined as a special disease. Since that time contributions on the subject of heaves have never been wanting. The statement of Funke ("Handb. d. Pathol. u. Therap. II. Aufl." Leipsig: 1850), that the various diseased conditions of heaves would in the future, when we became more experienced in the art of diagnosing, be recognized as a special

* From the Wochenschrift für Theirheilkunde und Viehzucht.

disease, has not been realized. It was not a fortunate proceeding for Gerlach, who deserves great merit for having given a scientific foundation to veterinary jurisprudence (" Gerichtl. Thierheilkunde," 1872) to admit of different forms of heaves. If heaves is not a special, but a whole group of diseases, different in their locality and their nature, it would not be logical to speak of different forms of heaves. I find that I can do justice to the demands of the warranty laws if I divide all the chronic non-febrile diseases and abnormal conditions giving rise to labored respiration, into three groups: 1. Abnormal conditions of the bronchi and lungs (Pulmonary Heaves); 2. Diseases of the heart (Cardiac Heaves); 3. Roaring (Laryngeal and Tracheal Heaves, etc.). The frequent assertions that heaves could be caused by hydrothorax (dropsical heaves of Gerlach), also by abnormal conditions of the diaphragm, especially diaphragmatic hernias, with a protusion of intestines into the thoracic cavity (asthma diaphragmaticum of old authors), and by diseased conditions of the abdominal organs, I do not find justifiable.

In discussing this subject I will confine myself to the first-named group (pulmonary heaves). That pulmonary heaves can be caused by various diseased conditions of the lungs, has long been admitted, without any plausible reasons having ever been produced. It would be erroneous to believe that the various forms of pulmonary heaves are always recognized by the symptoms of double expiration and by forced movements of the flanks in respiration. There are forms of pulmonary heaves, and I will mention them later, in which, even after hard labor, double expiration cannot be noticed, in which, rather in consequence of the hard labor, you could only observe a slight increase in the number of respirations, superficial movements of the flanks and an abnornal decrease in the number of respirations when bringing the horse to a halt. Those diseased conditions did not receive that consideration in literature that they were entitled to. I must admit they are not seen so frequently as other forms of heaves, that are so easily recognized by the symptoms of forced movements of the flanks and double expiration. Haubner, Gerlach, and other eminent veterinary authors, consider the last-named forms as being caused by "emphysema of the lungs." Gerlach asserts that abdominal respiration indicates the presence of emphysema. That this is not always the case is easily seen from the fact that in roaring, after hard labor, and in cases of hydrothorax, in which the lungs are compressed and contain but little air, also in cases of fatal internal hemorrhages, the labored respiration, with forced movements of the flanks (abdominal respiration), is the same as in emphysema. With the present condition of affairs, it seems to me that a critical discussion of the relations of pulmonary emphysema to heaves is indicated.

The word "emphysema" means inflation of an organ by the accumulation of gases in its substance or connective tissue. But, as in numerous other instances in pathology, the word "emphysema" has frequently been used independent of its original meaning. In connection herewith I will mention—although it does not properly belong to my subject—that the theory advocated by some authors, that in every case of emphysema you have an accumulation of *air* in the organs, does not prove to be correct. It is well known that, in pathological anatomy, a liver which has become inflated by the gases formed during the process of putrefaction is called emphysematous. In rinderpest and equine distemper, when the course of the disease has been protracted, you will find a general emphysema in isolated cases, which, owing to its mode of development, I have named "emphysema septicum." Quite recently I had a twelve-year-old gelding under treatment, suffering with paralysis of the intestines, a severe peritonitis and septicæmia, caused by a rupture of the rectum. Death ensued after six days. On the third day a slight emphysema of the sub-cutis was discovered on the right side of the neck. The day following the same condition was present in some places on the left side. Dyspnœtic symptoms were absent, and on making a post mortem examination I could not find a trace of interlobular emphysema. In this case, too, I consider that the emphysema was of a septic character. The fœtus of a cow dying intra-uterine, with the membranes still intact, will sometimes become so bloated with emphysema of the entire body, but especially of the sub-cutis, that it becomes impossible to deliver it. In milk fever of cows *(eclampsia parturientum)* you will occasionally find the tissue of the pia mater in the brain distended by the accumulation of gases, which originate in the blood and are forced through the walls of the capillaries by the circulation. Professor Harms was the first to discover this condition. The objection raised to this theory, that, by opening the head, air could have passed into the tissues of the brain, is without reason. Having made numerous post mortems on cows that died of milk fever, I must confirm Harms's statement, and claim the condition to be that of emphysema of the brain. However, I cannot endorse the assertion made by Harms, that the gases are atmospheric.

In numerous diseases I noticed that we can have a formation of gases in the lungs of a horse, the composition of which is not known, produced by the decomposition of inflammatory products or effused blood. The process of decomposition will extend from the original centre, gases entering into the interstitial and sub-pleural tissues to a greater or lesser degree. I have frequently diagnosed this form of pulmonary emphysema *intra vitam* by auscultation and percussion, in croupous pneumonia and pneumo-pleurisy, in catarrhal pneumonia, and in hæmorrhages of the lungs. It is in no way related to heaves. In one case the decomposition of the tissue and the formation of gases extended from the left lung, along the trachea, half way up the neck, within twenty-four hours before death. In connection with this, I wish to state that when this form of pulmonary emphysema is present you will do well to prognosticate an unfavorable result. Every case I observed terminated fatally.

Authors at the present time consider that an abnormal accumulation of atmosphere in the lungs is the cause of pulmonary emphysema. This theory originated with the eminent French pathologist, Laennec ("de la Auscultation Médiate, etc.," 1819), who was the first to use the word "pulmonary emphysema" in pathology. He was also the first to accomplish a separation of the terms "emphysema vesiculare" and "emphysema interstitiale seu lobulare."

After Laennec, in his classic work described "narrow-chestedness" (asthma) as pulmonary heaves, there was great danger of using this new theory in describing heaves in the horse. Long before this a number of veterinary authors asserted that the narrow-chestedness of man and of the horse must be caused by the same diseased condition. In fact, a number of respected French veterinarians did not hesitate to adopt this new theory in describing heaves. To a great extent, without giving the subject a thought, or examining into the conditions, they assumed that heaves was always caused by a rupture of the alveoli, and by the development of pulmonary emphysema. This unexplained interpretation was objected to by Hutrel d'Arboval, who, through his famous works ("Dictionnaire de Méd. et Chin. Vet.," 1826) influenced the practice of veterinary medicine for over ten years. He stated that he never succeeded in finding the cellular lung tissue filled with air, but always found the alveoli dilated in emphysema. In other respects, Hutrel coincided with the belief that heaves could be caused by numerous diseased conditions.

German veterinary authors mistrusted this new theory for a long time. After Rokitansky taught that vesicular pulmonary emphysema in man was caused by a loss of elasticity of the alveolar parenchyma (atrophy) and a complete disintegration of the alveolar septa (rarefaction des lungengewebes), in consequence of which several alveoli would unite and form a larger cavity, the theory apparently became more acceptable.

It is especially due to Bruckmüller, Haubner and Gerlach that the theory of vesicular and alveolar pulmonary emphysema being the cause of heaves was generally admitted by veterinarians.

For the past twelve years I have endeavored to settle the question of pulmonary emphysema to heaves, in consequence of which I made numerous post-mortem examinations. From my experience I can state that a general and extensive atrophy of the alveolar septa of the lungs, according to the opinion of Rokitansky, never occurs in horses. However, I frequently noticed that small portions of the lung were atrophied in consequence of a chronic diseased condition of the bronchi. This abnormal condition, which is frequently seen in old horses, does not cause respiration to become labored. Notwithstanding that small sections of the lung take no active part in respiration, the capacity of the lungs remains sufficiently large to supply the requisite amount of oxygen. I never found an extensive atrophy of the respiratory parenchyma, respiratory alveolar septa, or enlarged alveolar cavities caused thereby even after having carefully examined a large number of horses to decide this question. Consequently, I am obliged to question the existence of this form of pulmonary emphysema in horses.

Enlarged alveolar cavities (simple alveolarectasie) are frequent. While dying, life will sometimes cease when the act of inspiration is at its extreme limit, even when the horse was not troubled with heaves. A post-mortem reveals the fact that the lungs are filled with air to the extent of their capacity, remaining in this condition for hours, and will float if placed in water; the edges are somewhat rounded and the lungs feel "puffy," as old veterinarians frequently expressed it. I find an appropriate resemblance of touch in the feeling of a cushion moderately filled with feathers. The lungs readily show finger impressions, that disappear gradually. From a pathological anatomical standpoint it might be justifiable to describe this condition as a substantial pulmonary emphysema. But it is then necessary to consider that the condition is not one of disease.

Among small horses, shipped by rail from Russia to Berlin, I frequently had under treatment in the clinic some that were affected with a peculiar lung trouble ("Brust Krankheit"). In order to keep the freight charges low, owners are in the habit of overcrowding the cars. During the journey the horses are not only pitted against one another, but at times are thrown on top of one another. With all this trouble we find associated a constant chaffing, and detrimental drafts of cold air as a cause of disease. A few days following, we find the patient with acute pains in the thoracic cavity and a congestion of the internal organs, whilst we find the mucous membrane of the head, especially the conjunctiva, very pale and frequently anæmic in appearance. The cases generally develop into a circumscribed pneumonia or pleurisy, associated with a diminished peristaltic action of the intestines and a dilatory defecation. Owing to the symptoms which indicate severe pains in the wall of the chest, I have decided to call it "pleurodynia" (myalgia pectoralis) after a term used in human medicine. Horses will grunt when compelled to move, and after pressing the thorax, in consequence of the thoracic pains; respiration is increased from 40 to 50, at times 70 per minute and over, while the pulse generally remains normal, and only at times is increased to 55 per minute. The temperature taken per rectum is rarely elevated. The respiration is deep, with a superficial movement of the flanks, the walls of the chest being fixed, and in the lungs the condition develops, which the eminent pathologist Traube defined as "Volumen pulmonis acutum."

In this disease, which is not exclusively met with in horses that have been shipped, but is also at times produced from other causes, we get an inspiratory dilatation of the pulmonary alveoli, caused by the reduced action of expiration, on account of the severe pains in the walls of the thorax. Owing to the necessity of equalizing this insufficient respiration, the act of inspiration is performed to its extreme limit. But the act of respiration is not labored; on the contrary, there is an abnormal diminished movement of the flanks. Nor do any symptoms of abnormal respiration continue, excepting up to the time of death, in cases that terminate fatally, which usually occurs within two or three weeks, or four or six days. I have had the opportunity to examine numerous horses months after having recovered from pleuro-dynia, and found the respiration normal.

In cases of pulmonary heaves, a post-mortem examination generally, but not always, reveals a dilated lung which also is frequently seen in horses that have never been troubled with heaves. My experience has been, that in the majority of cases this dilated condition of the lungs could not be seen on post-mortem in horses that were afflicted with heaves. Therefore, in cases where this condition is present, it can only be due to the fact that death occurred at the moment of extreme inspiration. A general pulmonary emphysema accordingly has no relative connection with heaves, and it is erroneous to assert that it is the primary cause of heaves.

In horses that were afflicted with heaves, I frequently found on post-mortem examination a partial lobular emphysema. It is not essential to find this condition present, as in numerous cases of heaves (that are not well marked) it is not present. A partial pulmonary emphysema is not characteristic of heaves, as it is frequently found in acute inflammatory diseases of the lungs. Another cause is the increased pressure of inspired air on the lung tissue, to which some portions of the lung succumbs, because the surrounding diseased parts prevent a free and easy dilatation and contraction of the air passages. The development of a partial lobular emphysema, therefore, is due to a deficient respiratory capacity of some portions of the lungs, in consequence of which the pressure of the inspired air is unequally distributed, subjecting some portions of the tissue to an increased abnormal pressure. This increased pressure upon the walls of the alveoli is at times so great as to produce a permanent loss of elasticity. A partial emphysema can develop in any portion of the lungs; I have never discovered it in the central portion, but always on its periphery, most frequently on the edges and less so on the surface. The emphysematous portions are frequently the size of an egg and sometimes the size of a hand. In considering this question, I conclude from previous statements that a partial pulmonary emphysema is only complicated with the primary cause of heaves, and is not the *Ens morbi.*

It has been ascertained that interstitial or interlobular pulmonary emphysema in horses is only a form of heaves, and seldom occurs. Within the past thirty years numerous articles have been published in which it was stated, that after forced and unaccustomed labor of sound horses, a general interlobular and sub-pleural pulmonary emphysema was the result, which would never disappear. I feel satisfied that my observations in relation to the sudden appearance of this unusual form of heaves are in harmony with all statements made in literature. The history of the development of interlobular pulmonary emphysema is not thoroughly understood. It is evident that it must be preceded by a rupture of the walls of the alveoli or of the broncheoli. The rupture is undoubtedly very small, and owing to the contraction of the lungs after death, it becomes still smaller than it was *intra vitam.* It has been positively, but not frequently asserted, that a general pulmonary emphysema is incurable. The atmospheric equivalent is not very great, nor is it readily absorbed by the organs into which it has penetrated, but this is no reason why such a condition should be incurable. It is probable that in numerous cases the rupture of the alveolar parenchyma does not contract, and therefore the emphysema of the interlobular connective tissue and of the sub-pleural spaces maintains itself to such a degree, that the opposing force of the tissues permits the admission of air. In other cases the continuance of emphysema may be due to consecutive changes in the bronchi and broncheoli, that become irreparable.

The frequent occurrence of interlobular pulmonary emphysema in the lobes at the lower edges of the lungs is generally known. This is the result of suffocation during the last acts of respiration, and must be attributed to this cause, because at the time of death all portions of the lungs are no longer equally utilized for this purpose, in consequence of which there is a sudden increased pressure on some parts of the lung tissue, causing a rupture of the alveolar parenchyma or bronchioli. It is still a question to what extent a primarily diseased condition of the bronchi or bronchioli assists in the development of interlobular pulmonary emphysema. In numerous instances I have been enabled to prove that after heaves existed for a number of years, caused by a diseased condition of the bronchial tubes, interlobular pulmonary emphysema gradually developed in that vicinity to a moderate degree, and I believe that the diseased condition of the bronchi favored its development in consequence of the increased pressure of the air on the surrounding tissues.

Interlobular pulmonary emphysema is easily diagnosed when it exists to an extent to enable one to readily recognize an abnormal and labored respiration, or is situated at a point which can be reached by auscultation and percussion. Dry râles, such as whistling, hissing, etc., very prominent during the act of expiration, associated with pleuritic friction murmurs and a tympanitic sound on percussion, are symptoms that will readily enable a veterinarian to make a diagnosis. On the other hand, in considering the question whether, in concreto, interlobular pulmonary emphysema is in itself an absolute abnormality, or only a complication of a diffused bronchial ecstasy, or is in casual connection with putrid degenerating bronchopneumonic or haemorrhagic herds, is difficult to decide, and in some cases can only be decided after having the patient under observation for a few days. It is hardly necessary to mention that the temperature and other symptoms indicating a disturbance of health are of diagnostic value.

Herewith I have stated the relation of pulmonary emphysema to heaves in the horse. It now remains for me to describe the diseased conditions that cause heaves, or rather those that for practical forensic reasons should be considered as pulmonary heaves. So early as the last, and especially in the first half of the present century, veterinary authors recognized the fact that the word "heaves" included a whole group of diseases, and when I look over the works on pathology and monographic researches, I cannot help making honorable mention that some authors, notwithstanding the limited scientific experience of those days, have honestly endeavored to bring about a correct understanding of this subject. That they did not entirely succeed, may be attributed to the fact that the physiological conditions of normal respiration were not understood until a later period.

The necessity of excluding all inflammatory and febrile diseases of the lungs from the group of heaves was taught correctly in principle for a few score years. Even if the authors did not make it a thorough success in this respect, we must overlook it, because the nature of the exudative, suppurative and gangrenous diseases of the lungs were not sufficiently investigated, thermometry was unknown, and the method of examining by auscultation and percussion not thoroughly understood. To-day it is not necessary to make any remarks about the value of these aids in diagnosing.

With good judgment those authors only included such chronic and non-febrile diseases as would give rise to dyspnœa and cause death, not immediately, but only after a long time. They included:

1. Abnormalities, which greatly diminish the contractibility of the lungs, and thereby render expiration so difficult as to necessitate the auxiliary muscles of respiration to come into play;

2. Abnormal conditions, which partly obstruct the atmosphere from penetrating into the respiratory parenchyma;

3. Diseases, which greatly diminish the respiratory surface of the lungs.

This accordingly verifies the statement that the most important, and, I will add, most frequent, forms of heaves are due to diseased conditions of the bronchi and bronchioli. That a chronic inflammation of the bronchial tubes frequently gives rise to heaves, has been prominently mentioned, a long time ago, by the English veterinarian, White, and especially by the French veterinarian, Rodet, in his interesting monograph (*Memoire sur la Pousse*, Paris, 1828). It is to be regretted that Rodet mentions a long list of conditions that do not exist, supposed to develop from the inflammation of the bronchi, as being essential to the development of heaves. His labors would otherwise have been crowned with a more permanent success.

Ad. 1. The diseases of the bronchial tubes that produce the most common forms of heaves, are:

A. A diffused chronic bronchitis, associated with a general bronchial ecstasy. This form characterizes itself in most horses by a dry inflammation of the bronchi and bronchioli (bronchitis *chronica sicca diffusa*, or, as called by old-time authors, "asthma *siccum*"), and not seldom as a mucous catarrh of the bronchial tubes (bronchitis *chronica catarrhosa*, or "asthma *humidum*"). The disease produces a weakness and a paralysis of the muscular tissue imbedded in the walls of the bronchial tubes. Owing to this paralysis, we get an insufficient expiration, in consequence of which it becomes necessary for the horse to breathe with an abnormal frequency, in order to equalize the respiration. I previously mentioned that when this is carried on to an extreme limit it may produce a partial pulmonary emphysema. I will also mention that I have seen cases of diffused bronchitis and bronchial ecstasy which, after continuing for years, caused a development of tracheitis, as well as a gradual emaciation, cachexia and death.

B. Diffused chronic peribronchitis. As the name indicates, this disease runs its course in the peribronchial tissue. We could therefore consider it as bronchitis fibrosa externa. This disease is not so frequent as the chronic bronchial affection mentioned under "A." The cause of heaves in this case is also due to a paralysis of the bronchial muscles. We therefore have no difference in the dyspnœtic respiration from that mentioned under "A." On post-mortem, I found the lungs collapsed, flabby, and on cross section numerous grey-white spots, from the size of a lintel to that of a pea (thickened walls of the bronchi), without any distinct demarcation, surrounded by the flesh-colored lung tissue. From the centre of the grey-white spots I could at times press out a mucous secretion. The mucous membrane of the smaller bronchi and bronchioli was reddened and covered with a thin layer of viscid mucous.

Ad. 2. C. Another disease of the bronchial tubes in horses is one that affects the bronchial mucous membranes, and is recognized by thickening of the same. With a thickening of the mucous membrane, lining the smaller bronchi, there must be a deficient supply of air to the alveoli, in consequence of which we see an excessively increased respiration, associated with a superficial movement of the flanks, and a dilatation of the nostrils at hard labor. I consider this form of heaves as being caused by an endo-bronchitis seu brochiti interna chronica.

Ad. 3. Other diseased conditions that diminish the respiratory surface of the lungs, and are included under the term heaves, are :

D. Fatty degeneration of the lungs, a chronic indurating affection of the parenchyma of the lungs and its interstices, which appears in numerous herds, and at times conglomerated it will extend over a large portion of the lungs. This condition, as I stated in the *Wochenschrift für Thierheilkunde u. Viehzucht* in 1879, p. 14, represents a definite and characteristic disease of the lungs, that is probably caused by a peculiar infection, and the term indurated pneumonia, which term is applied to changes taking place in the simple connective tissue, and is therefore hardly applicable to this disease. I prefer the term "fatty pneumonia;" this term undoubtedly has the advantage of guarding one against mistakes. It is more frequently seen in old than in young horses. In numerous instances I had the opportunity to prove by post-mortem examinations that this disease was the cause of heaves. In such horses that have shown labored respiration for six months, one year or more, the fatty degeneration assumes extensive proportions, whereby the horse is rendered useless. It is incurable. The symptoms are increased respiration and a superficial movement of the flanks.

E. Tumors in the lungs. Frequently we have a formation of numerous secondary sarcomas in numerous herds, seldom a metastatic carcinoma. In gray horses I frequently found on post-mortem examination a melano-sarcoma in the lungs and on the pleura that had been the cause of heaves. If these malignant tumors are located in parts of the lung that are inaccessible by auscultation and percussion, their presence cannot be diagnosed. The symptoms are about the same as those described under D. (fatty pneumonia).

F. Absolute interlobular pulmonary emphysema. It was previously stated that this condition can arise after a single but excessive exertion, and continue as an incurable form of heaves.

In considering this subject it is not my object to go into further details of the etiology and symptomatology of the various forms of heaves. For practical reasons, however, I will make one more statement; I coincide with the opinion of Gerlach, because it has been his as well as my experience, that in order to positively certify as to the presence of heaves, it is necessary that the horse be examined *intra vitam.* From the results of the necroscopy alone, you cannot always prove that the animal was affected with a non-febrile disease during life, nor that the horse was troubled with a labored and increased respiration while at work. In giving an opinion about these conditions we must not forget that a large section of the lungs may be diseased and symptoms of abnormal respiration missing, also that the remaining healthy portion may supply the necessary amount of air for respiration.

